# Cancer métaplasique du sein: à propos d'un cas

**DOI:** 10.11604/pamj.2014.19.268.5508

**Published:** 2014-11-11

**Authors:** Moulay Abdellah Babahabib, Adil Chennana, Aymen Hachi, Jaoud Kouach, Driss Moussaoui, Mohammed Dhayni

**Affiliations:** 1 Service de Gynécologie Obstétrique, Hôpital Militaire d'Instruction Mohamed V, Rabat, Maroc

**Keywords:** carcinome, métaphasique, sein, carcinoma, Metaplastic, breast

## Abstract

Les carcinomes métaplasiques du sein sont des tumeurs rares. Ils constituent un groupe hétérogène de tumeurs définis selon l'organisation mondiale de la santé comme étant un carcinome canalaire infiltrant mais comportant des zones de remaniements métaplasiques (de type épidermoïde, à cellules fusiformes, chondroïde et osseux ou mixte), qui varient de quelques foyers microscopiques à un remplacement glandulaire complet. Les aspects cliniques et radiologiques ne sont pas spécifiques. Le traitement associe la chirurgie, la radiothérapie et la chimiothérapie. L'hormonothérapie n'a pas de place. Le pronostic est sombre. L'histopathologie combinée à l'immunohistochimie permet de poser un diagnostic sure. Etant donné que la prise en charge thérapeutique est limitée, une nouvelle approche moléculaire pourrait modifier cette contribution faible et mal cernée des traitements systémiques classiques. Les patientes atteintes de carcinome métaplasique mammaire pourraient bénéficier de traitements ciblés, ce qui reste à confirmer par des essais cliniques.

## Introduction

Les carcinomes métaplasiques du sein sont des tumeurs rares particulièrement intéressantes de la part de leur différence, clinique, radiologique, anatomopathologique et thérapeutique par rapport à ceux de la forme habituelle du cancer du sein. Les auteurs rapportent un nouveau cas de carcinome métaplasique du sein, et à travers l'analyse des données de la littérature, ile mettent le point sur les différents aspects de ce type de carcinome mammaire.

## Patient et observation

**Figure 1:**
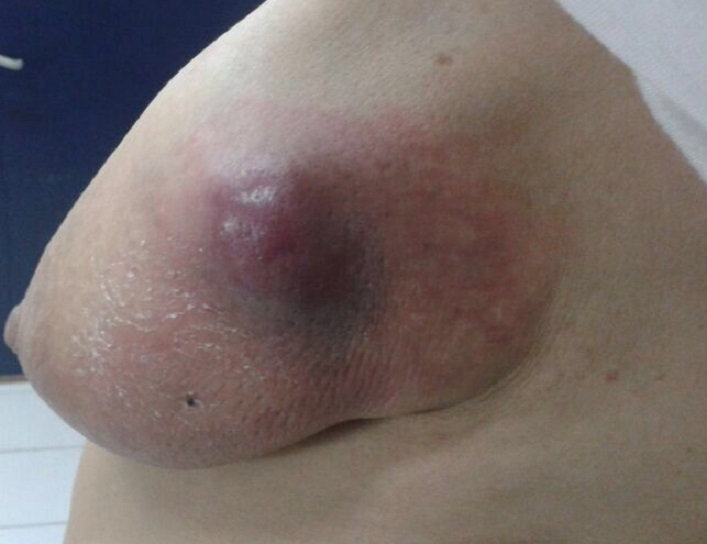
Tuméfaction inflammée du quadrant superoexterne du sein gauche

**Figure 2:**
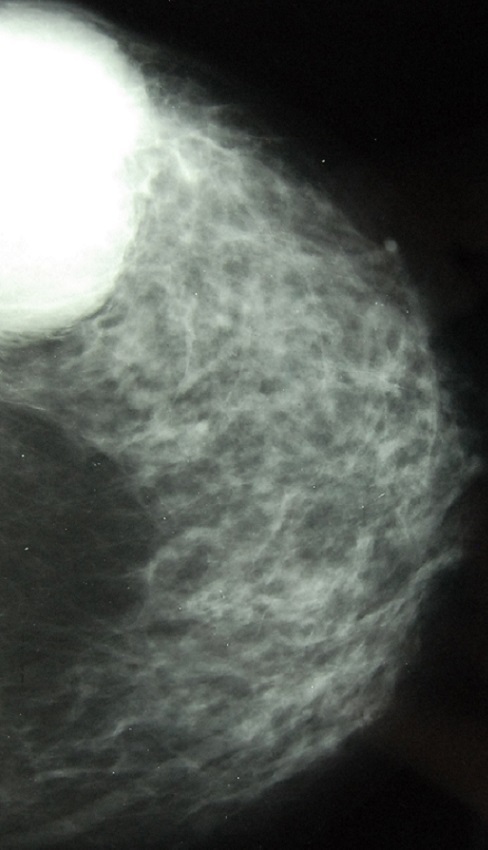
Mammographie cliché de face du sein gauche

**Figure 3:**
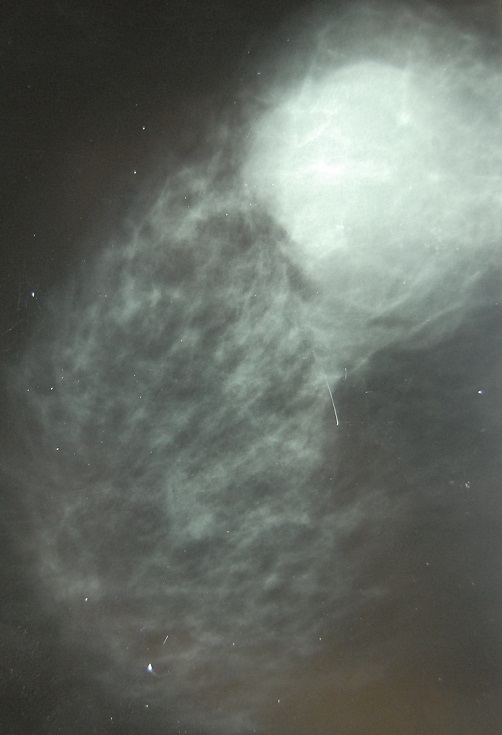
Mammographie cliché de profil du sein gauche

**Figure 4:**
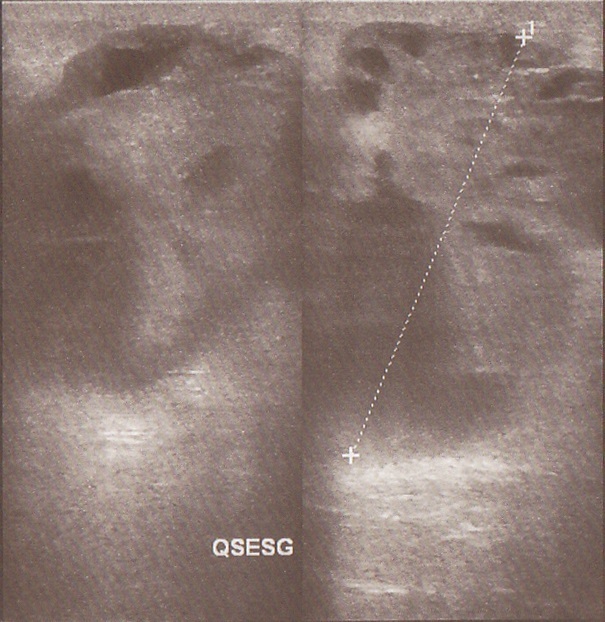
Aspect solido-kystique de la tumeur à l’échographie

Mme S.A, 62 ans, sans antécédent particuliers, ménopausée depuis 10 ans sans prise de traitement hormonal substitutif de la ménopause, hospitalisée en décembre 2013 à l'hôpital militaire d'instruction Mohammed V (HMIM-V) de Rabat pour une tumeur du sein gauche découverte à l'autopalpation. L'examen clinique trouve une tuméfaction rénitente du sein gauche avec une masse sous-jacente du quadrant supèro-externe faisant 6 cm/6 cm, mal limitée, dure, fixe et douloureuse associée à des signes inflammatoires à type de placard érythémateux et peau d'orange sans écoulement mammellonaire (Figure 1). L'examen des aires ganglionnaires axillaires et sus-claviculaires trouve une adénopathie axillaire homolatérale de 2cm/2cm fixe et indolore le reste de l'examen somatique est sans particularité. La mammographie trouve une opacité du quadrant supèro-externe de 6 cm de grand axe, de contours irréguliers sans foyers de micro calcifications (Figure 2). A l’échographie mammaire la lésion est de forme arrondie, de contours irréguliers, hétérogène, solide et kystique (Figure 3, Figure 4), vascularisée au Doppler couleur. Cet aspect écho-mammographique est classé BI-RADS 4 de l'ACR. Les microbipsies au tru-cut ont objectivé un carcinome galactophorique grade II invasif et nécrosé.

**Figure 5:**
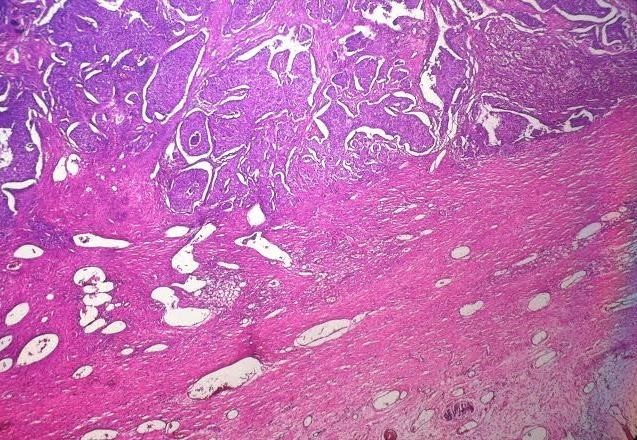
Coloration HE Gx10 prolifération tumorale

**Figure 6:**
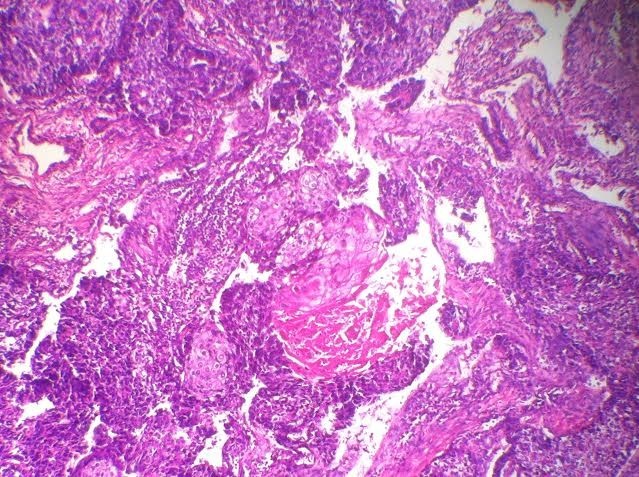
Coloration HE Gx20 foyer de différenciation malpighien

Le bilan d'extension incluant la radiographie du thorax, l’échographie hépatique et la scintigraphie osseuse a objectivé un nodule pulmonaire calcifié du lobe supérieur droit non spécifique qui a été confirmé par un scanner. Il s'agit donc d'un stade T3N1M0. Un traitement chirurgical radical avec curage ganglionnaire axillaire type Patey a été pratiqué. L'examen macroscopique de la pièce de mastectomie montre à la coupe une grosse tumeur assez bien limitée dure blanchâtre mesurant 5 cm de grand axe. Aux smears on trouve un processus malin de haut grade. L’étude histopathologique trouve une prolifération carcinomateuse partiellement nécrosée faite de cordons, de massifs de petits amas avec de rares tubes (score3).les cellules ont un noyau augmente de taille, anisocaryotique, hyperchromatique et nucléolé (score2).le cytoplasme est éosinophile. L'index mitotique est de 15 mitoses par 10 champs au fort grossissement (score3)(Figure 5).les cellules montrent des signes de différenciation malpighienne, avec des signes de dyskératose (Figure 6).

L'examen anatomopathologique a conclu en un carcinome metalpasique épidermoïde grade II de Scarff Bloom Richardson. Il n'a pas été vu de carcinome in situ, d'embols vasculaire, ou d'engainements péri veineux. Pas de maladie de Paget. Le plan profond passe à 0.1cm de la tumeur les autres limite sont saines et il n ya pas d'envahissement ganglionnaire, les récepteurs hormonaux et l'herceptest sont négatifs. La patiente est actuellement sous chimiothérapie 3 FEC(5-fluorouracile,Epirubicine Cyclophosphamide)100 + 3 taxotére, L’évolution avec un recul de deux mois n'a pas révélé de récidive ou de métastase.

## Discussion

Le carcinome métaplasique du sein est une tumeur maligne primaire rare mais en croissance [1, 2] représentant 0,2 5% des cancers du sein. Une croissance du nombre de cas rapporté annuellement a été notée et peut être expliquée par une augmentation de l'incidence de la maladie et l'attention accordée à son diagnostic [1, 2]. La classification de l'organisation mondiale de la santé 2003 distingue les carcinomes purement épithéliaux incluant les carcinomes épidermoïde, les adénocarcinomes avec différenciation fusiforme les carcinomes adénosquameux et les carcinomes mixte à double composante épithéliale et mésenchymateuse [3].

Ces tumeurs surviennent chez des femmes ménopausées avec un âge moyen de 53 ans [4] ce qui correspond à l’âge de notre patiente. La symptomatologie clinique est non spécifique. La tumeur se révèle généralement sous forme de masse comparable à une tumeur bénigne mais habituellement d’évolution rapide. Des mastodynies, des signes inflammatoires, un écoulement mamelonnaire, une rétraction du mamelon, des ulcérations de la peau en regard et parfois un abcès mammaire sont souvent rapportés. L'atteinte ganglionnaire est rare [5, 6]. Chez notre patiente l’évolution rapide de la tumeur, l'aspect abcédé et l'inflammation plaident plus en faveur de la malignité. L'aspect radiographique aussi est non spécifique. Mais des signes comme l'hyperdensité de la masse et l'absence de micro calcifications peuvent être évocateurs à la mammographie [5]. L’échographie mammaire peut mettre en évidence des foyers de remaniements hémorragiques, nécrotiques ou kystiques, qui sont fréquents [7]. Ces tumeurs peuvent échapper au dépistage car d’évolution habituellement rapide [4]. Chez notre patiente l’échographie a objectivé une masse arrondie, irréguliers, hétérogène, solide et kystique.

L'aspect macroscopique est non spécifique, il s'agit généralement d'une tumeur ferme, bien limitée, avec une taille tumorale importante variant entre 0,5 et 18 cm [4, 6]. Chez notre patiente la tumeur est da grande taille faisant 6 cm de grand axe. Sur le plan histologique, les carcinomes épidermoïdes sont de diagnostic facile, ils sont caractérisés par une prolifération de cellules malpighiennes polygonales reliées par des desmosomes apparents, avec ou sans foyers de dyskératose. L'adénocarcinome avec métaplasie à cellules fusiformes correspond à un carcinome glandulaire avec des foyers étendus à cellules fusiformes de nature épithéliale. Les carcinomes adénosquameux sont faits de deux contingents épithéliaux malins, glandulaire et épidermoïde. Les carcinomes métaplasiques mixtes sont caractérisés par l'association d'un carcinome infiltrant et des éléments mésenchymateux hétérologues représentés par des zones de différenciation cartilagineuse, osseuse ou musculaire... Lorsque le contingent mésenchymateux est malin, la tumeur est appelée carcinosarcome [8]. L'association à un cancer canalaire in situ n'est pas rare (50% da cas) [6]. Sur le plan immuno-histochimique, les récepteurs hormonaux sont positifs dans moins de 17% des cas [2] et la surexpression de HER2 aussi est souvent absente et les carcinomes métaplasiques mammaires sont triples négatifs dans 64% à 96% des cas [1]. L'atteinte ganglionnaire est rare et varie entre 6 et 26% [1]. Chez notre patiente la tumeur montre une différenciation malpighienne (Figure 5, Figure 6) avec présence de cellules dyskératosiques avec recepteurs hormonaux et hercept test négatif à l'immunohistochimie la fixation de la cytokeratine 5/6 n'a pas été recherchée, les ganglions étais tous négatifs.

L'histogenèse du carcinosarcome a été longtemps sujette à controverses. Actuellement, l'hypothèse la plus probable suggère la transformation phénotypique particulière de cellules épithéliales en cellules myoépithéliales, puis en sarcome [9]. Sur le plan biologie moléculaire, les carcinomes métaplasiques du sein présentent un profil transcriptomique de type basal et expriment un ou plusieurs marqueurs de type myoépithélial ou basal (p63, 34'E12, cytokeratine5/6, CK14, la protéine S100, l'actine et l'EGFR). Différentes études ont trouvé une surexpression d'EGFR Human Epidermal Growth Factor Receptor-1(HER1) qui pourrait suggérer une réponse favorable de ces tumeurs aux traitements ciblant EGFR (HER1) [10]. Les carcinomes métaplasique du sein posent d'importants problèmes de diagnostic différentiel essentiellement avec les tumeurs phyllodes et les sarcomes mammaires primitifs. En cas de métaplasie épidermoïde pure, il faut éliminer la possibilité d'un carcinome d'origine cutanée ou d'une métastase. Devant une tumeur à cellules fusiformes le principal diagnostic différentiel est la tumeur phyllode maligne [8]. Devant un carcinome avec métaplasie osseuse ou chondroïde on doit éliminer un fibroadénome, un ostéosarcome, un chondrosarcome et une tumeur phyllode. De nombreux types de sarcomes peuvent siéger au niveau mammaire, les plus fréquents étant l'angiosarcome et le liposarcome [10].

Le traitement repose sur la chirurgie. Elle est souvent radicale, mais un traitement chirurgical conservateur est possible pour les petites tumeurs [1]. Le curage ganglionnaire axillaire est recommandé, malgré leur caractère peu lymphophile [1]. Le rôle de la chimiothérapie et de la radiothérapie est encore discuté [7]. La radiothérapie adjuvante post-opératoire est peu indiquée car le traitement chirurgical conservateur est moins fréquent et les ganglions souvent négatifs [1], mais elle semble avoir un rôle essentiel dans le contrôle des récidives locales après le traitement chirurgical conservateur [10]. La chimiothérapie standard est non satisfaisante, car la chimiorésistance est fréquente [7]. L′hormonothérapie n'a habituellement pas de place, vu l'absence habituelle d'expression des récepteurs hormonaux. L'herceptine ne peut être introduite dans la plupart des cas, car l′Herceptest est souvent négatif. La surexpression d'EGFR(HER1), pourrait suggérer une réponse favorable de ces tumeurs aux traitements ciblant EGFR (anti HER1). D'autres thérapeutiques sont envisageables tel les sels de platine et les inhibiteurs de poly-ADP ribose polymérase (PARP) [10]. Le pronostic des carcinomes épidermoïdes reste péjoratif, le siège de prédilection des métastases survenant au cours des cinq premières années est le poumon, le foie, l'os ou le cerveau. La survie moyenne à 5ans est estimée entre 38 et 86%.

## Conclusion

Il est important d'identifier les carcinomes métaplasiques parmi les autres types du cancer du sein étant donné que leur prise en charge thérapeutique est différente et plus lourde. Le traitement de choix reste la chirurgie mais une nouvelle approche moléculaire pourrait modifier la contribution faible des traitements systémiques classiques.
